# BLong-term results of branch retinal vein occlusion: 5-year follow-up

**DOI:** 10.55730/1300-0144.6008

**Published:** 2025-03-15

**Authors:** İlkay KILIÇ MÜFTÜOĞLU, Natasha MAYER, Katherine DU, Catalina FEISTRITZER, Elise BARBERIS, Sashwanthi MOHAN, Jay CHHABLANI

**Affiliations:** 1 Department of Ophthalmology, İstanbul Training and Research Hospital, İstanbul, Turkiye; 2 Department of Ophthalmology, University of Pittsburgh Medical Center, Pittsburgh, PA, USA; 3 Department of Ophthalmology, Medcare Eye Centre, Al Safa, Dubai, United Arab Emirates; 4 Department of Education and Research, Rajan Eye Care, Chennai, India

**Keywords:** Anti-VEGF, branch retinal vein occlusion, macular edema

## Abstract

**Background/aim:**

This study aimed to report the 5-year outcomes of patients with newly diagnosed branch retinal vein occlusion (BRVO).

**Materials and methods:**

A retrospective review of patient charts was conducted. Data on central macular thickness (CMT), best-corrected visual acuity (BCVA), photoreceptor integrity, number of intravitreal (IV) antivascular endothelial growth factor (anti-VEGF) injections administered, and treatment regimen were assessed.

**Results:**

Ninety-seven eyes from individual patients with a mean age of 70.90 ± 11.49 (38–92) were included. The mean BCVA improved, and the mean CMT decreased at all follow-up visits following treatment (p < 0.05). Thirty-two eyes (33%) gained 3 lines of BCVA at the five-year follow-up. The mean number of total IV anti-VEGF injections administered was 19 ± 13.35 (1–49) during the five-year period, and 27.83% of patients received rescue laser treatment. Fifty-four percent of eyes received three consecutive monthly loading doses of IV anti-VEGF injections. The mean change in BCVA in the loading dose (+) group was significantly greater than in the loading dose (−) group at 1-year and subsequent visits. The loading dose (+) group showed a significantly greater reduction in CMT compared to the loading dose (−) group. The mean estimated time for BCVA to decline by ≥3 lines was 117 ± 8.74 months (95% CI = 100.21–134.51)

**Conclusion:**

Anti-VEGF treatment was effective in improving anatomical and functional outcomes in BRVO patients during long-term follow-up. Initial treatment with three loading doses of anti-VEGF resulted in greater vision gain and more pronounced anatomical improvement.

## 1. Introduction

Macular edema (ME) occurs in 5%–15% of branch retinal vein occlusion (BRVO) eyes within 1 year from the initial onset [1]. In untreated symptomatic BRVO patients, overall best-corrected visual acuity (BCVA) was poor at baseline, ranging from 20/40 to <20/200. Though BCVA shows some improvement over time, clinically significant improvement beyond 20/40 is uncommon in untreated eyes [2, 3, 4].

In the extension of the BRAVO study, the mean improvement from baseline BCVA letter score was 18.3 letters in the 0.5 mg ranibizumab group and 12.1 letters in the sham/0.5 mg ranibizumab group at 12 months [5, 6]. Seventy-six percent of these eyes were then enrolled in an open-label extension study (HORIZON) for another 12 months [7]. At month 12 of HORIZON, the mean change from BRAVO baseline was 15.6 and 17.5 letters in the sham/0.5 mg and 0.5 mg ranibizumab groups, respectively, which was not significantly different. Another open-label extension study (the RETAIN study), with a mean follow-up of 49 months [8], enrolling only 34 eyes from BRAVO, found that 50% of eyes had edema resolution after the last injection, whereas the other 50% still required an average of three ranibizumab injections during their final year of follow-up. In the VIBRANT study [9], aflibercept was found to be superior in terms of BCVA improvement compared to grid laser (+17 letters vs +6.9 letters at week 24, and +17.1 letters vs 12.2 letters at week 52).

Despite the presence of the studies mentioned above [5,8], data on the long-term prognosis of BRVO are limited due to the low number of patients included and the varying duration of follow-up. Moreover, clinical trials with strict inclusion–exclusion criteria and close monitoring do not reflect real-world data. Therefore, we conducted the largest long-term real-world study enrolling 97 BRVO patients, aiming to report their functional and anatomical outcomes and to identify factors associated with long-term visual acuity prognosis.

## 2. Materials and methods

This was a retrospective, longitudinal study comprising patients who were newly diagnosed with BRVO and followed for a minimum of 5 years between 2012 and 2022 at a single referral center. Institutional Review Board approval was acquired for the review and analysis of patient medical records and related images. The study adhered to the tenets of the Declaration of Helsinki for research involving human subjects and complied with the Health Insurance Portability and Accountability Act (HIPAA) regulations.

The diagnosis of BRVO was made based on ophthalmological examination by an experienced retina specialist (JC), and ME was confirmed using spectral-domain optical coherence tomography (SD-OCT) (Cirrus 5000, Carl Zeiss Meditec). Inclusion criteria were defined as follows: a minimum of five years of follow-up, absence of accompanying vitreoretinal diseases that may bias the results, such as age-related macular degeneration, history of retinal artery occlusion, or diabetic retinopathy. The patients’ medical records were retrieved from electronic charts. Age, sex, systemic diseases, smoking status, preexisting ocular diseases, and lens status were noted.

### 2.1. Ophthalmological examination and retinal imaging

The patients underwent visual acuity (VA) assessment (by pinhole or manifest refraction), slit-lamp biomicroscopy, intraocular pressure measurement, and dilated fundus examination, along with SD-OCT imaging, at all follow-up visits. Ischemia was assessed clinically, and fundus fluorescein angiography (FA) was performed when needed.

The accuracy of delineating the appropriate layer on SD-OCT was confirmed by two masked observers (NM, KU). Central macular thickness (CMT) was obtained. Manual calipers were used to measure the CMT at the foveal center, with measurements performed independently by two reviewers. In cases of discrepancy between the reviewers, a third senior ophthalmologist reviewed the measurements to reach a consensus. This rigorous approach aimed to minimize observer variability and enhance the reliability of the CMT data. Subfoveal choroidal thickness (SFCT) was measured using the caliper function of the SD-OCT device, as previously described [11].

The integrity of photoreceptors was assessed at baseline and follow-up visits. The percentage of disruption in the external limiting membrane (ELM) and ellipsoidal inner segment (ISe) layer was measured along both the horizontal and vertical axes in the central 1000 μm and then averaged, as previously reported [12]. Here, “0%” represents no disruption, and “100%” represents complete loss of the layer. The presence of a foveal cyst and neurosensory detachment (NSD) was assessed and recorded as either ‘present’ or ‘absent’, without assigning a reference value.

Middle layer hyperreflectivity was defined as a hyperreflective band at the level of the inner nuclear layer (INL), with or without extension into the adjacent inner plexiform layer (IPL) and the outer plexiform layer (OPL) [13].

### 2.2. Treatment

In the presence of ME with accompanying decreased vision, intravitreal (IV) anti-VEGF injection [bevacizumab (Avastin, Genentech, US), ranibizumab (Lucentis, Genentech, US), aflibercept (Eylea, Regeneron, NY)] was considered. The choice of anti-VEGF agent was influenced by availability, insurance approval, and physician discretion. Patients were treated with IV injections using an OCT-guided as-needed approach. The treatment decision and the dosing interval for the injection were based on the clinician’s discretion and OCT guidance. The patients were divided into three groups based on whether a loading dose regimen was administered: loading dose (+) group, loading dose (−) group, and incomplete loading dose group. The loading dose (+) group received three initial consecutive monthly anti-VEGF injections after diagnosis; the incomplete group did not complete this initial regimen. Intravitreal steroid was administered, when necessary—particularly in resistant macular edema—in the form of triamcinolone acetate (TA) (Kenalog, Merck & Co., Inc., NJ, USA) or a dexamethasone implant (Ozurdex, Allergan, Inc., Irvine, CA). Laser treatment was also considered, at the physician’s discretion, in addition to anti-VEGF and/or corticosteroid therapy.

### 2.3. Statistical analysis

Continuous variables were presented as mean ± standard deviation (SD), and categorical variables were expressed as percentages (%). The distribution of the data was assessed using the Kolmogorov–Smirnov test. Snellen BCVA was converted to logMAR. The mean BCVA, CMT, SFCT, the extent of disruption in the ELM and ISe layers, the percentage of eyes with NSD or foveal cysts, inner and middle retinal hyperreflectivity, and INL thinning were reported at baseline and at specified follow-up visits. The mean changes in BCVA, CMT, SFCT, and the percentage of ISe and ELM layer disruption among the three groups were compared using one-way ANOVA with Bonferroni post hoc analysis or the Kruskal–Wallis test with Dunn’s post hoc analysis, depending on the normality of the data. The number of intravitreal injections at each follow-up visit was recorded. The necessity of laser treatment was also evaluated. In the case of missing data, the overall number of available data points was noted. A Kaplan–Meier survival plot was generated to estimate the time until a loss of 3 lines in vision [14]. SPSS software (IBM Corp., version 20.0, Armonk, NY, USA) was used for statistical analysis.

## 3. Results

Ninety-seven eyes of 97 patients, with a mean age of 70.90 ± 11.49 (range: 38–92), were included in the study. Sixty-one patients (62.88%) were female. Most of the patients (84.53%) were white. Seventy-four percent of patients were smokers. Hypertension (77.31%) was the most common systemic disease, followed by dyslipidemia (62.88%) and diabetes mellitus (30.92%). The mean interval between symptom onset and presentation was 29.08 ± 38.30 days (range: 1–180 days). Baseline demographics are given in Table 1.

**Figure 1:**
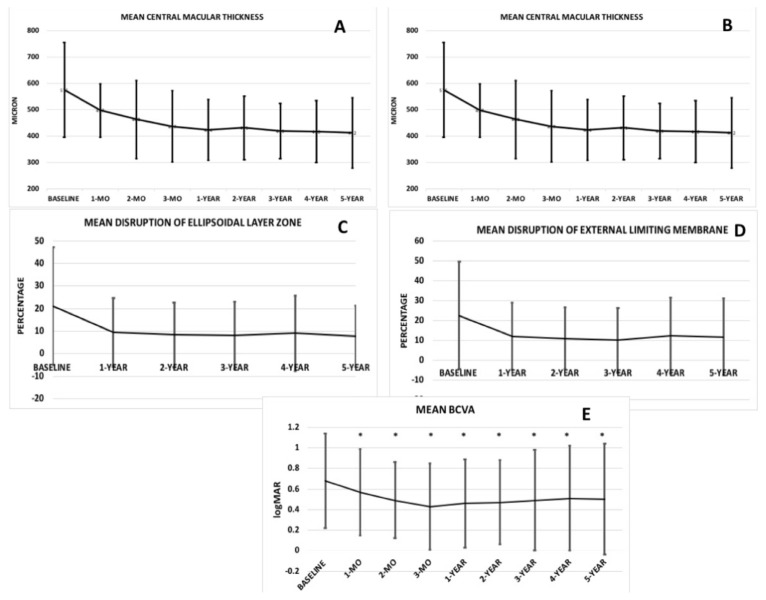
Anatomical SD-OCT parameters over follow-up.

**Figure 2:**
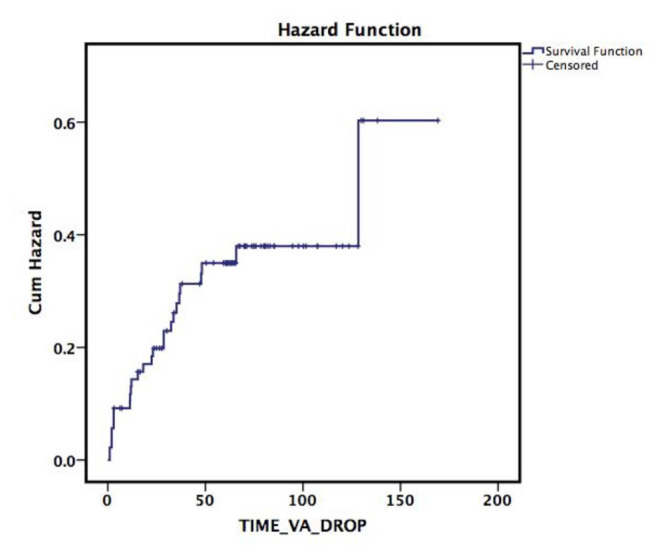
Survival plot for BCVA to drop ≥3 lines.

The mean CMT was 575.09 ± 180.21 μm (range: 265–1149 μm) at baseline in the study population. Compared to baseline, the mean CMT showed a statistically significant improvement at all visits during the 5-year follow-up (p < 0.001; Figure 1a). The mean SFCT remained stable over the 5-year follow-up, despite fluctuations observed at the 3-month visit (p = 0.70; Figure 1b). The mean percentage of disruption in the ISe and ELM layers was 21.0 ± 26.0% (range: 0–98%) and 22.5 ± 27.0% (range: 0–98%) at baseline, respectively. The integrity of the ISe and ELM layers significantly improved during follow-up (p < 0.001 for both layers, respectively; Figures 1c and 1d). The mean BCVA was 0.68 ± 0.46 logMAR (range: 0–2.0) (Snellen Equivalent » 20/95 ± 20/57; range: 20/20–20/2000) at baseline and 0.50 ± 0.54 logMAR (Snellen Equivalent » 20/63 ± 20/69; range: 20/20–20/2000) at the final follow-up. Compared to baseline, the mean BCVA showed statistically significant improvement from the 1-month visit through the 5-year follow-up (p = 0.021; Figure 1e). The percentages of patients who gained 3 lines of BCVA were 24.73% at 1 year, 31.32% at 2 years, 32.46% at 3 years, 28.9% at 4 years, and 32.35% at 5 years of follow-up. The distribution of BCVA over follow-up is given in Table 2. The mean estimated time for BCVA to drop by 3 lines was 117 ± 8.74 months (95% CI = 100.21–134.51; Figure 2).

The distributions of patients with NSD, foveal cysts, retinal layer hyperreflectivity, and INL thinning over the 5-year follow-up are provided in Table 3. As expected, compared to baseline, the percentage of patients with foveal cysts decreased at all visits (p = 0.043). The presence of NSD showed significant improvement starting from the 3-month follow-up (p = 0.003). Middle layer hyperreflectivity did not show a significant change over the follow-up period (p = 0.12). The inner nuclear layer showed statistically significant thinning starting from the 2-year visit through the 5-year visit.

### 3.1. Treatment

**Figure 3:**
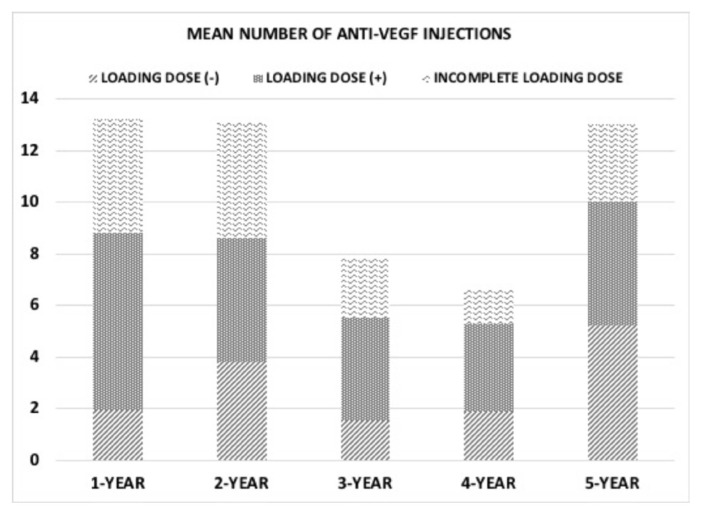
Mean number of anti-VEGF injections given based on the treatment regimen.

At baseline, 62 patients (63.9%) had ME. Approximately half of the patients (54.6%) received three monthly loading doses of IV anti-VEGF injections. Seventeen eyes (15.7%) did not receive any loading regimen, whereas 27 eyes (27.8%) initially received anti-VEGF injections but did not complete the loading regimen. The mean number of anti-VEGF injections was 2.27 ± 1.41 (range: 0–4) at 3 months, 5.38 ± 3.19 (0–12) at 1 year, 5.11 ± 2.99 (0–11) at 2 years, 3.12 ± 3.43 (0–10) at 3 years, 2.57 ± 3.02 (0–11) at 4 years, and 4.53 ± 2.70 (0–10) at 5 years. The mean total number of IV anti-VEGF injections was 19 ± 13.35 (range: 1–49) during the 5-year follow-up in the overall study population. More specifically, the total number of anti-VEGF injections was 33.86 ± 10.26 (range: 9–49) in the LD (+) group, 26.11 ± 7.57 (16–38) in the incomplete LD group, and 19.75 ± 9.40 (9–33) in the no LD group; the LD (+) group had significantly more anti-VEGF injections than the LD (−) group (p = 0.003). Data on anti-VEGF injections by loading dose group are illustrated in Figure 3.

During the 5-year follow-up, a total of 27.83% of patients received laser treatment (2.0% at 3 months, 10.30% at 1 year, 7.20% at 2 years, 2.0% at 3 years, 5.1% at 4 years, and 1.0% at 5 years). A total of eight eyes received IV steroid injections in the form of TA or a dexamethasone implant. The mean number of total IV steroid injections was 2.4 ± 1.8.

### 3.2. Treatment response

**Figure 4:**
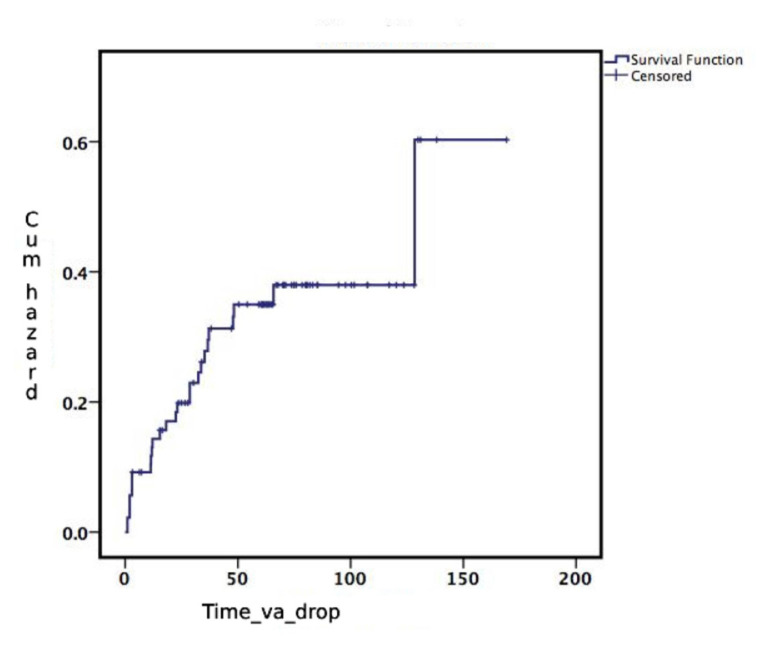
Mean change in anatomical and functional parameters based on the treatment regimen. (mo: months)

Starting from the 3-month visit, the loading dose (+) group showed a significantly greater decrease in CMT compared to the loading dose (−) group (−191 μm vs −28 μm, p = 0.003 at 3 months; −190 μm vs −72 μm, p = 0.017 at 1 year; −211 μm vs −64 μm, p = 0.014 at 2 years; −222 μm vs −72 μm, p = 0.018 at 3 years; −208 μm vs 85 μm, p = 0.046 at 4 years; −234 μm vs 114 μm, p = 0.05 at 5 years). A significant difference in the mean change in CMT was observed between the loading dose (+) group and the incomplete loading dose group only at the 3-month visit (p = 0.029; −191 μm vs −27 μm). The most prominent decrease in CMT was observed at the 5-year follow-up visit across all groups (Figure 4a).

The mean change in SFCT was not statistically significant at any visit during the follow-up period among the three groups (p = 0.56; Figure 4b). The ISe and ELM layers showed a greater extent of restoration in the loading dose (+) group than in the loading dose (−) group at all follow-up visits. The mean change in ISe layer disruption between the loading dose (+) and loading dose (−) groups was statistically significant at 1 year (p = 0.003), 2 years (p = 0.004), 3 years (p = 0.001), 4 years (p = 0.011), and 5 years (p = 0.026) of follow-ups. No significant difference was observed in the mean change in disruption of the ellipsoidal layer between the loading dose (+) group and the incomplete loading dose group (Figures 4c and 4d).

**Figure 5:**
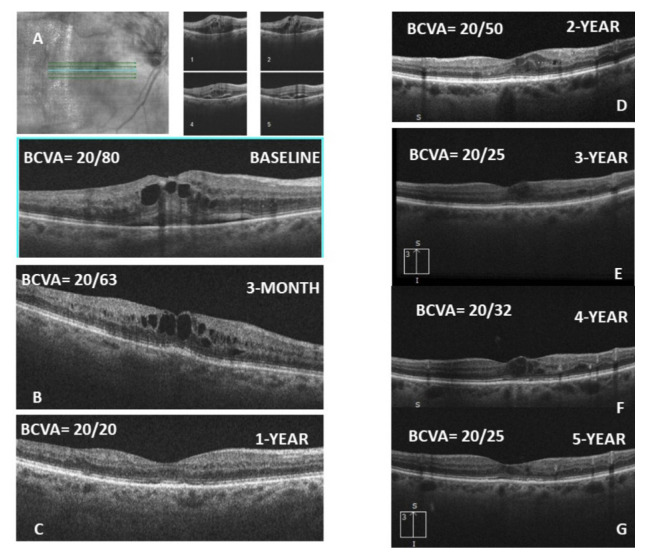
A 62-year-old female with a BCVA of 20/80 was presented with superior branch retinal branch occlusion in the right eye (A). She was initially treated with three loading doses of bevacizumab injections (B). Over 5 years, she received a total of 18 anti-VEGF injections (C, D, E), and the BCVA increased to 20/25 at the last visit.

The loading dose (+) group tended to show a greater increase in BCVA compared to the other two groups at all follow-up visits (Figure 4e). The mean change in BCVA in the loading dose (+) group was significantly higher than in the loading dose (−) group at the 1-, 2-, 3-, 4-, and 5-year follow-up visits (mean difference in BCVA change between the two groups: »22 letters, p = 0.036; »21 letters, p = 0.016; »20 letters, p = 0.010; »18 letters, p = 0.033; »17 letters, p = 0.04, respectively). There was no significant difference in the mean BCVA change between the loading dose (+) and the incomplete loading dose (−) group at any follow-up visit. (Figure 5)

## 4. Discussion

In this real-world study involving 97 treatment-naïve BRVO patients, the mean BCVA began to improve at the 1-month follow-up visit and was maintained throughout the 5-year follow-up. The patients also demonstrated significant anatomical improvement, including a reduction in central macular thickness and restoration of photoreceptor layer integrity.

The mean BCVA was 20/100 at baseline and improved to 20/60 at the final follow-up, with a slight decline starting from the 3-month follow-up. Twenty seven percent of eyes had good vision (20/40) at baseline, which increased to 50.7% at the final follow-up, whereas the percentage of eyes with poor vision remained stable at the 5-year follow-up. At 5 years, 32.35% of eyes gained 3 lines of BCVA, which is consistent with findings from other studies [10,15]. Conversely, during the 5-year follow-up, 27 eyes showed a loss of more than three lines of BCVA at some follow-up visits. The mean time for vision to drop by ^3^3 lines was as long as 117 months. Ischemia and/or progressive photoreceptor damage might be responsible for the vision loss observed during long-term follow-up.

In a recent systematic review [15], at 5 years, BCVA improved from 55.4 letters to 71.7 letters, and CMT decreased from 496 to 181, suggesting that sustained benefits are possible for at least 5 years with ongoing treatment. Another real-world study [10] reported a vision gain of +11 letters at 1 year, and %34 of eyes achieved a 15-letter improvement after 1 year. At 5 years, the majority of patients improved or maintained vision, with 22% gaining 15 ETDRS letters.

In a study involving 28 eyes of 28 BRVO patients [16], the authors reported that the number of anti-VEGF injections decreased over time during the 5-year follow-up, and only two eyes required further treatment beyond 2 years. Although eyes treated with IVR or IVB showed significant improvement in anatomical and functional outcomes at the 1-year visit, only the IVR group maintained the long-term benefit of treatment. Conversely, another real-world study [10] reported the mean number of anti-VEGF injections administered as 6.9 ± 3.3 at 1 year, and that the injection frequency did not decline in the subsequent years. At 5 years, a mean of 28.4 ± 16.6 injections was administered. In BRVO-related ME, a decrease in the frequency of injections may not be as evident as in diabetic macular edema, although the development of collaterals over time may help reduce the need for continuous injections. In our study, the number of injections given at 1 year was almost the same as at year five (5.38 ± 3.19 vs 4.53 ± 2.70). In the RETAIN study, which had a mean follow-up of 50.2 months [8], 50% of BRVO patients experienced resolution of edema; however, the other half of the eyes still required an average of three ranibizumab injections during their final year of follow-up.

A study found that 56.35% of BRVO patients and 42.5% of CRVO patients showed resolution of ME at the 3-year visit, with fewer injections required in each year of follow-up [17]. The proportion of eyes with a BCVA of ≥20/40 was 43.6% at 6 months, 39.2% at 1 year, 39.4% at 2 years, and 42.2% at the 3-year follow-up [17]. However, reporting the combined results (BRVO+CRVO) may introduce bias and may not allow for a head-to-head comparison with our study.

When categorizing the eyes based on the loading dose regimen, improvements in CMT and restoration of photoreceptor layer integrity were consistently more prominent in eyes receiving the loading dose regimen throughout the 5-year period. Moreover, this translated into greater visual gain over the 5-year follow-up. On the other hand, having an incomplete or no loading dose regimen did not appear to affect visual gain, despite a trend toward improved vision in the incomplete LD group.

The study has limitations such as its retrospective nature, use of various IV anti-VEGF agents, lack of a strict treatment algorithm that might cause bias, and absence of fundus fluorescein angiography images in the long-term. Due to the retrospective feature of our study from real-world data, our choice of anti-VEGF agent was influenced by factors including availability, insurance approval, and physician discretion. Therefore, showing the effect of one type of anti-VEGF agent over long-term follow-up would not be possible. On the other hand, considering the overlap between systemic diseases in the long-term, no significant correlation was observed with any specific systemic condition. In a small number of cases with mostly persistent ME (ranging from 2.75% to 4.5%), IV corticosteroids and laser treatment were used. However, due to the very low number of eyes that received these treatments, we were not able to perform further analysis to determine the individual effect of each treatment.

In conclusion, visual acuity can improve with ongoing anti-VEGF treatment in BRVO patients starting from the first injection, and this improvement might be maintained over long-term follow-up. The loading dose regimen may yield better anatomical and functional outcomes. One-third of eyes gained 3 lines of BCVA over long-term follow-up, which is a promising outcome. Further studies comparing different anti-VEGF agents are needed to better understand the potential superiority of individual IV agents. In addition, long-term studies with sustained delivery agents are necessary to determine whether longer treatment-free intervals can be achieved.

## Figures and Tables

**Table 1:** Baseline characteristics of the study population.

Age, mean ± SD (range), years	70.90 ± 11.49 (range: 38–92)

Sex, n (female/male)	61/36

Race, n (%)	
White	82 (84.53%)
African-American	15 (15.46%)

Smoking status yes, n (%)	72 (74.22%)
Low	38 (52.77%)
Moderate	30 (41.66%)
Heavy	6 (8.33)

Systemic diseases, present, n (%)	
Coronary artery disease	20 (20.61%)
Hypertension	75 (77.31%)
Diabetes mellitus	30 (30.92%)
Dyslipidemia	61 (62.88%)

Lens status, n	

Pseudophakia/phakia	27/97

**Table 2:** Distribution of eyes with varying BCVA over 5-year follow-up.

	20/40	20/40–20/200	£20/200
**Baseline**	27%	55.2%	17.7%
**1-month**	33%	54%	12.6%
**2-month**	37.5%	50%	12.5%
**3-month**	45.3%	44%	10.6%
**1-year**	28.7%	59.5%	14.6%
**2-year**	45.7%	40.9%	13.25%
**3-year**	45.4%	42.8%	11.6%
**4-year**	52.1%	31.8%	15.94%
**5-year**	50.7%	32.8%	16.4%

**Table 3:** Distribution of SD-OCT parameters over follow-up.

	NSD (+)	Foveal cyst (+)	Inner layer hyperreflectivity (+)	Middle layer hyperreflectivity (+)	INL thinning (+)
**Baseline**	51.54%	92.78%	87.62%	78.35%	1.00%
**1-mo**	45.71%	n/a	n/a	n/a	n/a
**2-mo**	44.82%	n/a	n/a	n/a	n/a
**3-mo**	32.14%*	n/a	n/a	n/a	n/a
**1-year**	28.57%*	80.21%*	89.13%	82.60%	3.29%
**2-year**	25.88%*	77.64%*	84.70%	77.64%	8.23%*
**3-year**	27.84%*	75.94%*	84.50%	77.21%	8.45%*
**4-year**	22.53%*	78.87%*	81.17%	71.83%	9.85%*
**5-year**	25.35%*	76.05%*	88.73%	69.01%	11.39%*

NSD: neurosensorial detachment, INL: inner nuclear layer, mo: month.

* p < 0.05
